# Eupafolin enhances TRAIL-mediated apoptosis through cathepsin S-induced down-regulation of Mcl-1 expression and AMPK-mediated Bim up-regulation in renal carcinoma Caki cells

**DOI:** 10.18632/oncotarget.11604

**Published:** 2016-08-25

**Authors:** Min Ae Han, Kyoung-jin Min, Seon Min Woo, Bo Ram Seo, Taeg Kyu Kwon

**Affiliations:** ^1^ Department of Immunology, School of Medicine, Keimyung University, Dalseo-Gu, Daegu 704-701, South Korea

**Keywords:** eupafolin, TRAIL, Mcl-1, cathepsin S, AMPK

## Abstract

Eupafolin, a flavone found in *Artemisia princeps*, has been reported for its anti-tumor activity in several cancer cells. In this study, we examined whether eupafolin could sensitize TRAIL-mediated apoptosis in human renal carcinoma Caki cells. We found that eupafolin alone and TRAIL alone had no effect on apoptosis. However, combined treatment with eupafolin and TRAIL markedly induced apoptosis in human renal carcinoma (Caki) cells, glioma cells (U251MG), and prostate cancer cells (DU145), but not normal cells [mesangial cells (MC) and normal mouse kidney cells (TCMK-1)]. Eupafolin induced down-regulation of Mcl-1 expression at the post-translational levels in cathepsin S-dependent manner, and over-expression of Mcl-1 markedly blocked apoptosis induced by combined treatment with eupafolin and TRAIL. In addition, eupafolin increased Bim expression at the post-translational levels via AMP-activated protein kinase (AMPK)-mediated inhibition of proteasome activity. Knock-down of Bim expression by siRNA inhibited eupafolin plus TRAIL-induced apoptosis. Furthermore, combined treatment with eupafolin and TRAIL reduced tumor growth in xenograft models. Taken together, these results suggest that eupafolin enhanced TRAIL-mediated apoptosis via down-regulation of Mcl-1 and up-regulation of Bim in renal carcinoma Caki cells.

## INTRODUCTION

Eupafolin (6-methoxy 5, 7, 30, 40-tetrahydroxyflavone) is a flavone extracted from the *Artemisia princeps*. It has been widely used by Chinese or native Indians as traditional medicine [1]. Eupafolin exhibits multiple functions including anti-inflammatory, anti-oxidant and anti-proliferative effects [2–5]. Recently, it has been reported that eupafolin induces apoptosis in human cervical adenocarcinoma cells through induction of mitochondrial membrane depolarization [3]. Eupafolin suppresses proliferation and growth of prostate cancer cells through inhibition of PI3K activity [4]. Molecular mechanisms underlying the anti-cancer effects of eupafolin can be associated with activation of caspase [3], down-regulation of anti-apoptotic proteins [3] and the inhibition of Akt signaling pathway [4]. However, the exact mechanism of anti-cancer effect of eupafolin remains elusive.

Tumor necrosis factor-related apoptosis-inducing ligand (TRAIL) is the member of the TNF ligand family, and it has been shown to be effective in inducing apoptosis in variety tumor cells, but not normal cells [6, 7]. Binding of TRAIL to death receptor (DR) 4 and 5, allows the recruitment of FADD and caspase-8, which results in the formation of the death-inducing signal complex (DISC) [8]. In addition, TRAIL also triggers the mitochondrial apoptotic pathway through disruption of the mitochondrial membrane permeability resulting in the releases of cytochrome *c* into the cytosol [9]. Although TRAIL selectively induces cancer cell death, the down-regulation of pro-apoptotic proteins and death receptors and up-regulation of anti-apoptotic proteins such as c-FLIP, Bcl-2, Bcl-xL and inhibitor of apoptosis proteins (IAPs) lead to resistance to TRAIL-induced apoptosis [10–14]. Therefore, there are several limitations to using the TRAIL or the TRAIL receptor antibody. The short half-life of human soluble TRAIL, inherent TRAIL resistance and acquired TRAIL resistance by repeated TRAIL exposure are considered to be the limitation factors. Therefore, identification of TRAIL sensitizers is needed to overcome the hurdle of TRAIL resistance.

In this present study, we investigated whether the TRAIL resistance in cancer cells could be overcome by combination treatment with eupafolin and TRAIL. Moreover, the molecular mechanisms underlying eupafolin plus TRAIL-induced apoptosis was further investigated.

## RESULTS

### Effect of eupafolin on TRAIL-mediated apoptosis in human renal carcinoma Caki cells

**Figure 1:**
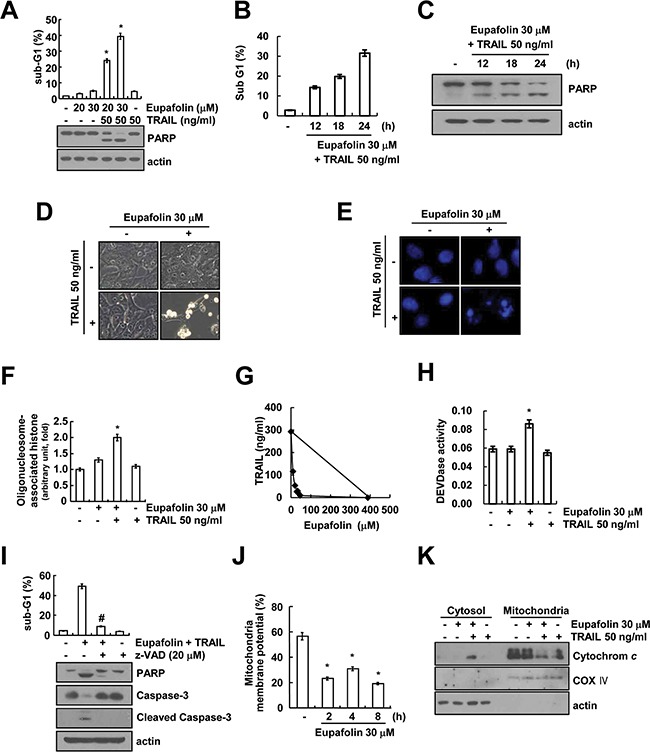
Eupafolin sensitizes TRAIL-induced apoptosis in Caki cells **A-C.** Caki cells were treated with 50 ng/ml TRAIL in the presence or absence of the indicated concentrations with eupafolin for 24 h (A) or indicated time periods (B and C). Apoptosis was analyzed as a sub-G1 population by flow cytometer. The protein levels of PARP and actin were determined by Western blotting. The level of actin was used as a loading control. **D-F.** Caki cells were treated with 50 ng/ml TRAIL in the presence or absence of 30 μM eupafolin for 24 h. Cell morphology was detected by interference light microscopy (D). The condensation and fragmentation of the nuclei were detected by 4′, 6′-diamidino-2-phenylindole staining (E). The DNA fragmentation detection kit determined the fragmented DNA (F). **G.** Isoboles were obtained by plotting the combined concentrations of each drug required to produce 50% cell death. The straight line connecting the IC_50_ values obtained for the two agents when applied alone corresponded to the addition of their independent effects. Values below this line indicate synergy, whereas values above this line indicate antagonism. **H.** Caki cells were treated with 50 ng/ml TRAIL in the presence or absence of 30 μM eupafolin for 24 h. Caspase activities were determined with colorimetric assays using caspase-3 DEVDase assay kits. **I.** Caki cells were treated with 30 μM eupafolin plus 50 ng/ml TRAIL for 24 h in the presence or absence of 20 μM z-VAD-fmk (z-VAD). The sub-G1 fraction was measured by flow cytometry. The protein expression levels of PARP, caspase-3, cleaved caspase-3 and actin were determined by Western blotting. The level of actin was used as a loading control. **J.** Caki cells were treated with 30 μM eupafolin for the indicated time periods and loaded with a rhodamine123 fluorescent dye. The mitochondrial membrane potential (MMP) was measured using a flow cytometer. **K.** Caki cells were treated with 50 ng/ml TRAIL in the presence or the absence of 30 μM eupafolin for 24 h. Cytoplasmic fractions were analyzed for cytochrome c release. The level of COX IV was used as a mitochondria loading control. The level of actin was used as a loading control. Apoptosis was analyzed as a sub-G1 population by FACS. The values in (A, B, F, H, I and J) represent the mean ± SD from three independent samples. * p < 0.05 compared to the control. # p < 0.01 compared to the combined treatment with eupafolin and TRAIL.

Because eupafolin has anti-tumor activity in several cancer cells [3, 4], we investigated whether eupafolin could sensitize TRAIL-mediated apoptosis in human renal carcinoma Caki cells. First, Caki cells were treated with eupafolin alone (20, 30 μM), TRAIL alone (50 ng/mL), and combined treatment with eupafolin and TRAIL. Apoptosis was determined using flow cytometric and Western blotting. As shown in Figure 1A, combined treatment with eupafolin and TRAIL markedly induced accumulation of sub-G1 population and cleavage of PARP, whereas treatment with eupafolin alone and TRAIL alone had no effect on apoptosis. In addition, combined treatment with eupafolin and TRAIL also induced accumulation of sub-G1 population and cleavage of PARP in a time dependent manner (Figure 1B and 1C). Combined treatment with eupafolin and TRAIL also induced apoptotic characteristics such as apoptotic body formation, cell shrinkage, and cell detachment on the plate (Figure 1D). Next, we analyzed nuclear condensation and DNA fragmentation, which is the hallmark of apoptosis. Eupafolin plus TRAIL-induced the DNA fragmentation and the nuclear condensation (Figure 1E and 1F). Moreover, combined treatment with various concentrations of eupafolin and TRAIL showed synergistic effects (Figure 1G). Next, we examined whether caspase activation plays a critical role in eupafolin plus TRAIL-induced apoptosis. Eupafolin plus TRAIL increased caspase-3 activity (Figure 1H), but not caspase−2, −8 and −9 (Supplementary Figure S1). The activation of caspase-3, accumulation of sub-G1 population and cleavage of PARP were completely prevented by pre-treatment with the pan-caspase inhibitor, z-VAD-fmk (Figure 1I). It has been known that the loss of mitochondria membrane potential (MMP) plays a critical role in apoptosis by releasing cytochrome *c* into the cytoplasm [15]. Therefore, we examined whether the loss of MMP is involved in eupafolin plus TRAIL-induced apoptosis, by using rhodamine123 fluorescence dye. As shown in Figure 1J, eupafolin markedly reduced MMP levels, and increased cytosolic cytochrome *c* release in combined treatment with eupafolin and TRAIL (Figure 1K). These results suggest that combined treatment with eupafolin and TRAIL can induce caspase-dependent apoptosis in Caki cells.

### Effects of eupafolin on expression levels of apoptosis-related proteins

**Figure 2:**
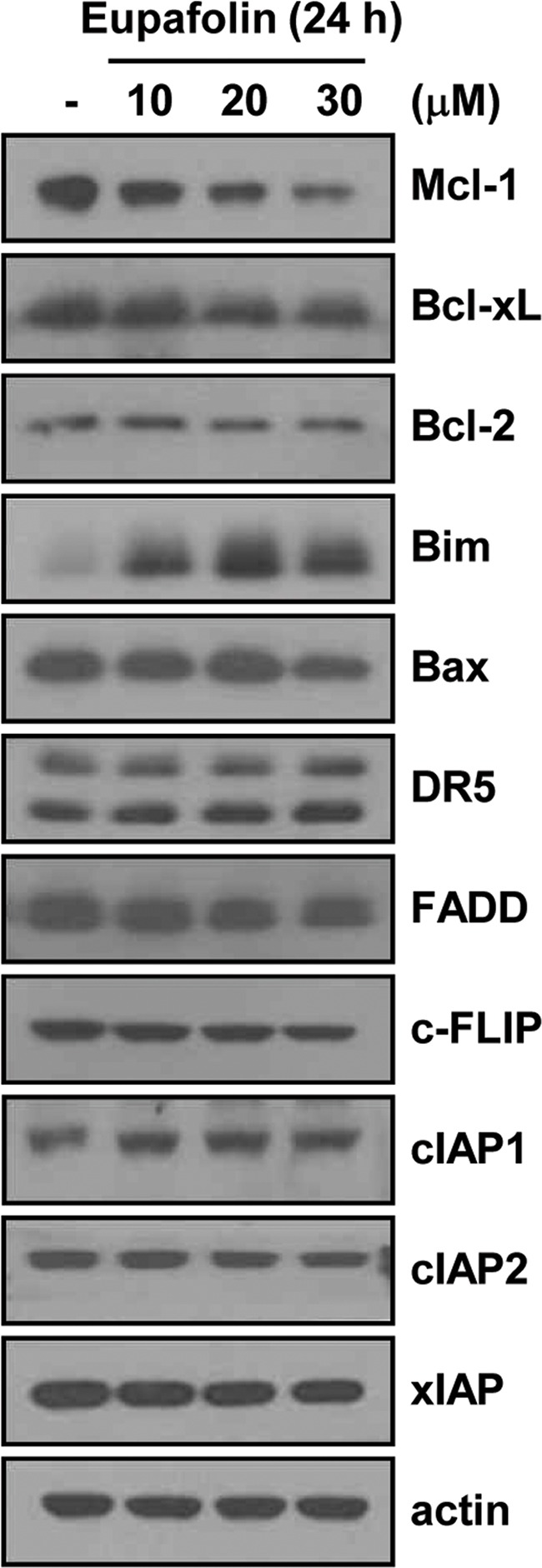
Effects of eupafolin on expression levels of apoptosis-related proteins Caki cells were treated with the indicated concentrations of eupafolin for 24 h. The protein levels of Mcl-1, Bcl-xL, Bcl-2, Bim, Bax, DR5, FADD, c-FLIP, cIAP1, cIAP2, XIAP, and actin were determined by Western blotting. The level of actin was used as a loading control.

Next, to determine the molecular mechanisms underlying eupafolin-mediated TRAIL sensitization, we investigated expression levels of apoptosis-related proteins. Eupafolin markedly induced down-regulation of Mcl-1 expression, and up-regulation of Bim expression (Figure 2). In contrast, levels of other apoptosis-related proteins were not altered in response to eupafolin.

### Eupafolin induces down-regulation of Mcl-1 expression at the post-translational level

**Figure 3:**
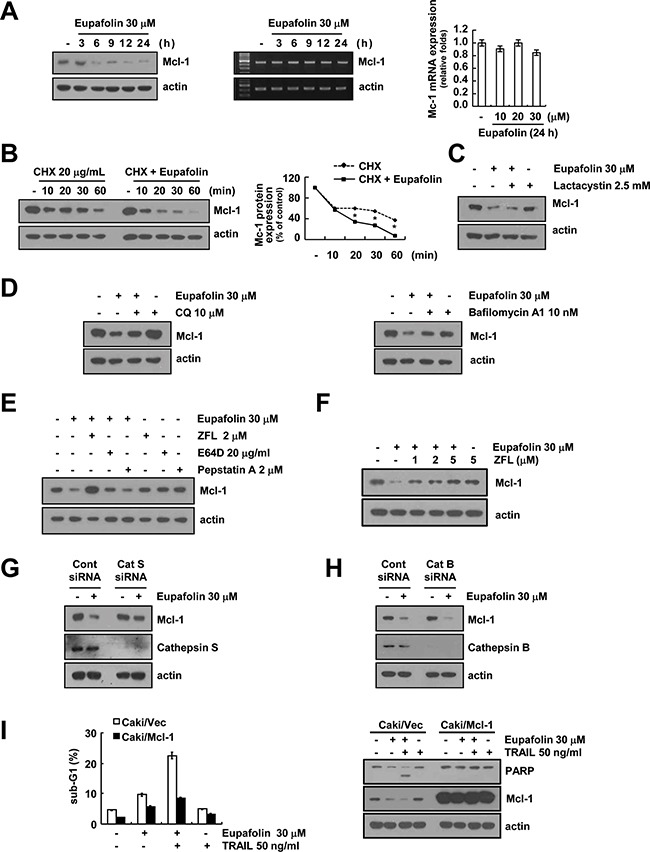
Eupafolin induced cathepsin S-mediated down-regulation of Mcl-1 expression **A.** Caki cells were treated with 30 μM eupafolin for the indicated time periods. The protein and mRNA levels of Mcl-1 were determined by Western blotting (left panel), RT-PCR (middle panel) and real-time PCR (right panel), respectively. The level of actin was used as a loading control. **B.** Caki cells were pre-treated with 20 μg/mL cycloheximide (CHX) for 30 min, and then treated with 30 μM eupafolin for the indicated time periods. The protein levels of Mcl-1 were determined by Western blotting. The level of actin was used as a loading control. The band intensity of the Mcl-1 protein was measured using ImageJ (public domain JAVA image-processing program; http://rsb.info.nih.gov/ij, lower panel). **C.** Caki cells were pre-treated with lactacystin 2.5 μM for 30 min, and then treated with 30 μM eupafolin for 24 h. The protein levels of Mcl-1 and actin were determined by Western blotting. The level of actin was used as a loading control. **D.** Caki cells were pre-treated with chloroquine (CQ, 10 μM) and bafilomycin A (10 nM) for 30 min, and then added 30 μM eupafolin for 24 h. The protein levels of Mcl-1 and actin were determined by Western blotting. The level of actin was used as a loading control. **E.** Caki cells were pretreated with the 2 μM ZFL, 20 μg/ml E64D, and 2 μM pepstatin A for 30 min, and then added with 30 μM eupafolin for 24 h. The protein levels of Mcl-1 and actin were determined by Western blotting. The level of actin was used as a loading control. **F.** Caki cells were pretreated with the indicated concentrations of ZFL for 30 min, and then added with 30 μM eupafolin for 24 h. **G** and **H.** Caki cells were transiently transfected with cathepsin S (Cat S, G), cathepsin B (Cat B, H) siRNA or control siRNA. After transfection, cells were treated with 30 μM eupafolin for 24 h. The protein levels of Mcl-1, cathepsin B, cathepsin S, and actin were determined by Western blotting. The level of actin was used as a loading control. **I.** Vector cells (Caki/vector) and Mcl-1-overexpressed cells (Caki/Mcl-1) were treated with 50 ng/mL TRAIL in the presence or absence of 30 μM eupafolin for 24 h. Apoptosis was analyzed as a sub-G1 population by FACS. The protein levels of PARP and actin were determined by Western blotting. The level of actin was used as a loading control. The values in panel (B) represent the mean ± SD from three independent samples. * p < 0.05 compared to CHX.

To further determine the potential mechanisms underlying the eupafolin-induced down-regulation of Mcl-1 expression, we examined whether Mcl-1 expression is regulated at the transcriptional levels. Eupafolin reduced Mcl-1 protein expression within 6 h, but mRNA expression of Mcl-1 did not alter (Figure 3A). Because eupafolin had no effect on Mcl-1 mRNA expression, we subsequently examined the protein stability of Mcl-1. Caki cells were treated with cycloheximide (CHX, 20 μg/mL), an inhibitor of protein biosynthesis, in the presence or absence of eupafolin for various time points. As shown in Figure 3B, CHX alone gradually reduced Mcl-1 expression, but combined treatment with CHX and eupafolin more rapidly reduced Mcl-1 proteins expression. Since Mcl-1 is mainly degraded by the ubiquitin-proteasome pathway [16, 17], we tested whether proteasome-mediated Mcl-1 protein degradation is occurred in eupafolin-treated cells. Proteasome inhibitor (lactacystin) did not block eupafolin-induced down-regulation of Mcl-1 expression (Figure 3C). Next, to investigate whether Mcl-1 degradation was dependent on lysosome-degradation pathway, Caki cells were treated with inhibitor of lysosome function or lysosomal enzyme in the absence or presence of eupafolin. Chloroquine (CQ: lysosomotropic agent) and bafilomycin A (vacuolar ATPase inhibitor) inhibited eupafolin-mediated down-regulation of Mcl-1 expression (Figure 3D). Since cathepsin has a key role on lysosomal-mediated proteasome degradation pathway [18], we further investigated whether degradation of Mcl-1 expression was caused by cathepsins. Cathepsin S inhibitor (Z-FL-COCHO) and cathepsin B inhibitor (E64D) rescued eupafolin-mediated down-regulation of Mcl-1 expression, but not cathepsin D inhibitor (Figure 3E). In addition, cathepsin S inhibitor inhibited eupafolin-induced Mcl-1 down-regulation in a dose dependent manner (Figure 3F).

To further confirm that cathepsin S and B has an important role on down-regulation of Mcl-1 expression in eupafolin-treated cells, Caki cells were transiently transfected with cathepsin S and B siRNA, respectively. However, knock-down of cathepsin B by siRNA had no effect on eupafolin-induced down-regulation of Mcl-1 expression, but knock-down of cathepsin S by siRNA markedly inhibited Mcl-1 down-regulation caused by the treatment with eupafolin (Figure 3G and 3H). These results indicate that eupafolin-induced down-regulation of Mcl-1 expression was regulated at the post-translational levels in a cathepsin S-dependent manner.

To investigate the importance of the down-regulation of Mcl-1 protein in eupafolin plus TRAIL-induced apoptosis, we used Mcl-1-overexpressing cells. Combined treatment with eupafolin and TRAIL induced apoptosis in Caki/vector cell, but the induction of apoptosis and PARP cleavage by combined treatment with eupafolin and TRAIL markedly blocked in Mcl-1-overexpressing cells (Figure 3I). These data suggest that the down-regulation of Mcl-1 expression plays a critical role in combined treatment with eupafolin and TRAIL-induced apoptosis.

### Eupafolin induces up-regulation of Bim expression at the post-translational levels

**Figure 4:**
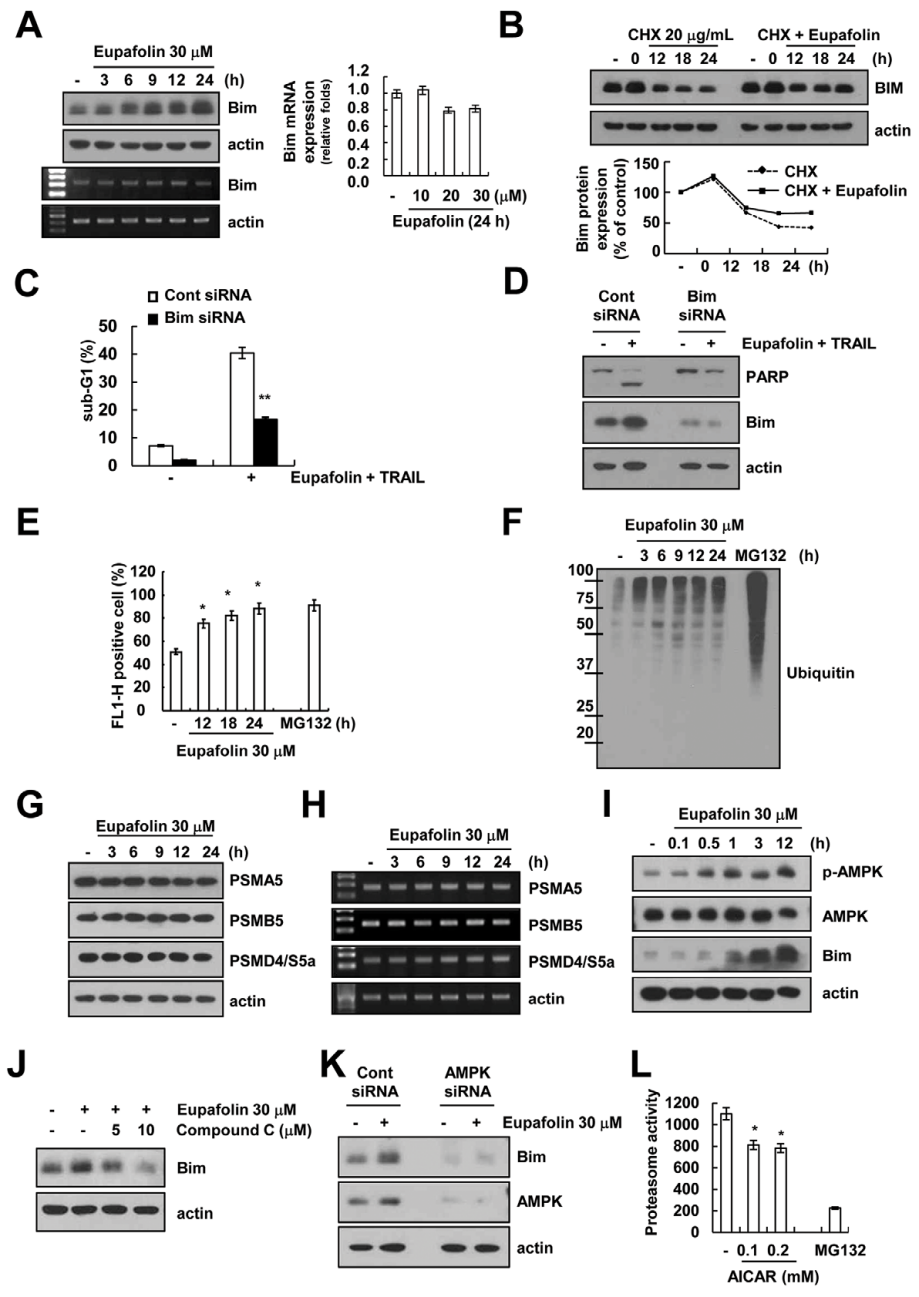
Eupafolin induces up-regulation of Bim expression through AMPK-mediated inhibition of proteasome activity **A.** Caki cells were treated with 30 μM eupafolin for the indicated time periods. The protein and mRNA levels of Bim were determined by Western blotting, RT-PCR and real-time PCR, respectively. The level of actin was used as a loading control. **B.** Caki cells were treated with 30 μM eupafolin for 12 h, and then cells were washed with PBS. Caki cells treated with 20 μg/mL CHX in the presence or absence of 30 μM eupafolin for the indicated time points. The protein levels of Bim and actin were determined by Western blotting. The level of actin was used as a loading control. The band intensity of the Bim protein was measured using ImageJ (public domain JAVA image-processing program; http://rsb.info.nih.gov/ij, lower panel). **C** and **D.** Caki cells were transiently transfected with Bim siRNA or control siRNA. Overnight after transfection, cells were treated with 30 μM eupafolin and 50 ng/ml TRAIL for 24 h. The level of apoptosis was analyzed by measuring the sub-G1 fraction by flow cytometry. The protein levels of PARP, Bim, and actin were determined by Western blotting. The level of actin was used as a loading control. **E.** Caki/ZsGreen cells were treated with 1 μM MG132 (as a positive control) and indicated concentrations of eupafolin for 24 h. Proteasome activity was analyzed by using FACS analysis. **F.** Caki cells were treated with 30 μM eupafolin for the indicated time periods (MG132: positive control). The protein levels of ubiquitin were determined by Western blotting. **G** and **H.** Caki cells were treated with 30 μM eupafolin for the indicated time periods. The protein levels of PSMA5, PSMB5, PSMD4/S5a, and actin were determined by Western blotting (G) and RT-PCR (H), respectively. The level of actin was used as a loading control. **I.** Caki cells were treated with the 30 μM eupafolin for the indicated time periods. The protein levels of phospho(p)-AMPK, AMPK, Bim, and actin were determined by Western blotting. The level of actin was used as a loading control. **J.** Caki cells were pretreated with the indicated concentrations with compound C for 30 min, and then added 30 μM eupaforin for 24 h. The protein levels of Bim and actin were determined by Western blotting. The level of actin was used as a loading control. **K.** Caki cells were transiently transfected with AMPK siRNA or control siRNA. Overnight after transfection, cells were treated with 30 μM eupafolin for 24 h. The protein levels of Bim, AMPK and actin were determined by Western blotting. The level of actin was used as a loading control. **L.** Caki cells were treated with 1 μM MG132 (as a positive control) and indicated concentrations of AICAR for 24 h. The cells were lysed, and the proteasome activity was measured as described in the Materials and Methods. The values in panel (C, E and L) represent the mean ± SD from three independent samples. ** *p* < 0.05 compared to eupafolin plus TRAIL-treated control siRNA. * *p* < 0.05 compared to the control.

As shown in Figure 2, eupafolin up-regulated Bim expression levels. Therefore, we investigated the molecular mechanisms of eupafolin-mediated up-regulation of Bim expression. Eupafolin markedly induced Bim protein expression within 9 h, but Bim mRNA expression was not induced by treatment with eupafolin (Figure 4A). Therefore, we investigated whether eupafolin modulates the protein stability of Bim. Caki cells were treated with eupafolin for 12 h, washed with PBS, and then treated with or without eupafolin in the presence of 20 μg/mL cycloheximide (CHX) for the various indicated times. Eupafolin increased Bim protein stability in Caki cells (Figure 4B). To investigate the significance of the up-regulation of Bim expression, Caki cells were transiently transfected with Bim siRNA. The down-regulation of Bim expression by siRNA markedly inhibited eupafolin plus TRAIL-induced apoptosis (Figure 4C and 4D). These data indicated that Bim up-regulation plays a critical role on combined treatment with eupafolin and TRAIL-induced apoptosis.

Previous studies reported that Bim is mainly degraded by ubiquitin-proteasome system [19]. Therefore, we examined whether eupafolin could modulate proteasome activity. Analysis of fluorescence-based proteasome activity using ZsGreen (proteasome sensor vector) showed that eupafolin inhibited proteasome activity (Figure 4E), and increased ubiquitination in a time-dependent manner (Figure 4F). Next, we checked expression levels of three critical proteasome subunits: 20S proteasome subunit alpha type 5 (PSMA5), 20S proteasome subunit beta type 5 (PSMB5) and 19S proteasome non-ATPase regulatory subunit 4 (PSMD4/S5a) [20]. However, eupafolin had no effect on expression levels of three subunits (Figure 4G and 4H).

Recently, other groups reported that activation of AMP-activated protein kinase (AMPK) is associated with Bim expression [21, 22]. Therefore, we investigated whether activation of AMPK signaling is involved in eupafolin-induced Bim expression. First, we checked the effect of eupafolin on phosphorylation of AMPK. Eupafolin markedly induced AMPK phosphorylation within 0.5 h (Figure 4I). Furthermore, compound C (AMPK inhibitor) inhibited eupafolin-induced up-regulation of Bim expression in a dose dependent manner (Figure 4J). To further confirm the importance of AMPK signaling on eupafolin-induced Bim expression, Caki cells were transiently transfected with AMPK siRNA. Knock-down of AMPK by siRNA markedly inhibited eupafolin-induced Bim expression (Figure 4K). These data indicated that eupafolin induced up-regulation of Bim expression via activation of AMPK signaling. Next, we investigated whether AMPK signaling could modulate proteasome activity. Noticeably, proteasome activity is inhibited by 5-Aminoimidazole-4-carboxamide ribonucleotide (AICAR), AMPK activator (Figure 4L). Therefore, our data suggested that eupafolin induced up-regulation of Bim expression via AMPK-mediated inhibition of proteasome activity.

### Eupafolin sensitizes to other cancer cells to TRAIL-mediated apoptosis, but not normal cells

**Figure 5:**
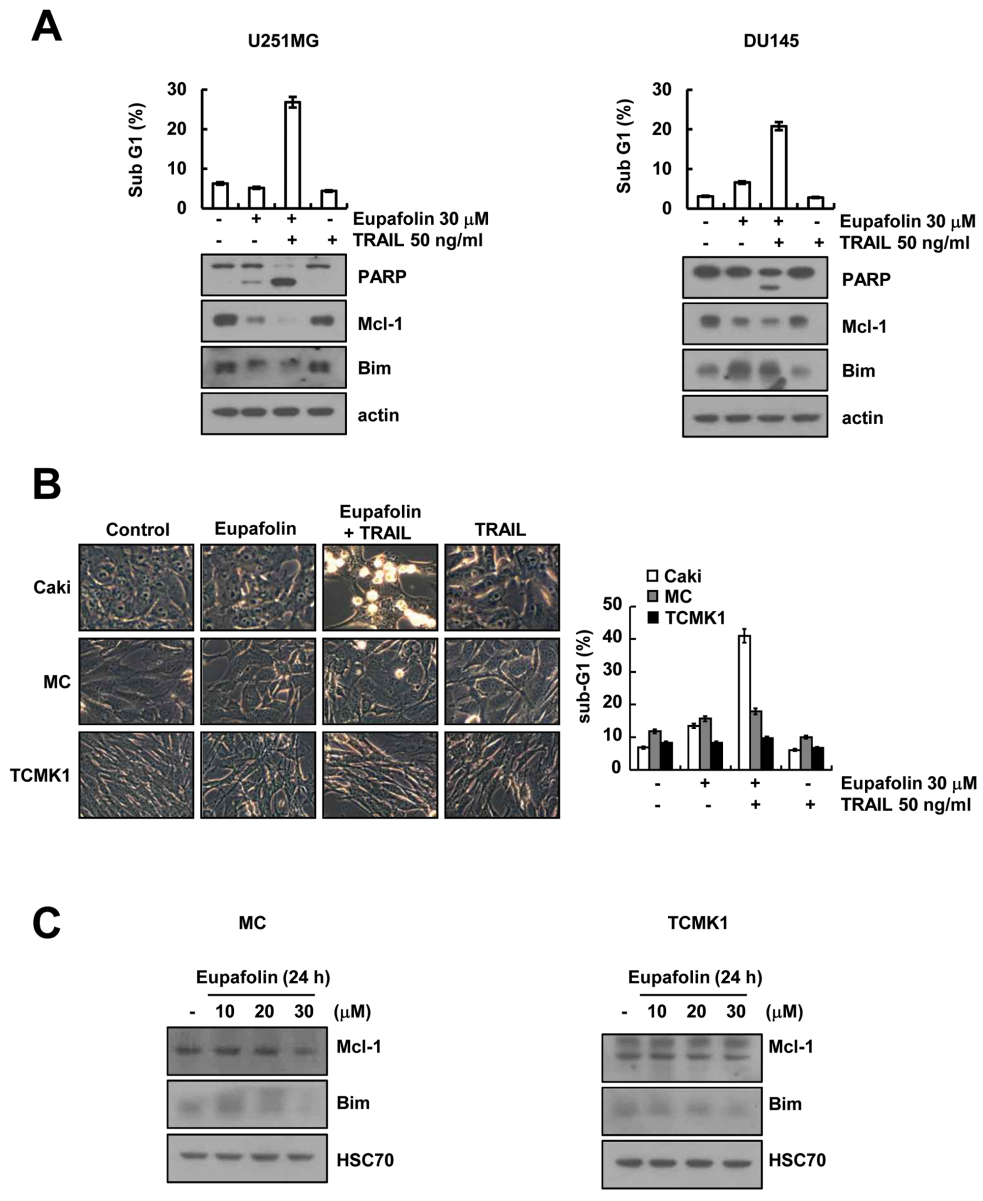
Effect of eupafolin plus TRAIL treatment on apoptosis of other carcinoma cells and normal cells **A.** U251MG (glioma cells) and DU145 cells (prostate cancer cells) were treated with 50 ng/ml TRAIL in the presence or absence of 30 μM eupafolin for 24 h. The level of apoptosis was assessed by measuring the sub-G1 fraction using flow cytometry (upper panel). The protein levels of PARP, Mcl-1, Bim, and actin were determined by western blotting. The level of actin was used as the loading control (lower panel). **B.** Caki, mesangial cells (MC) and mouse renal tubular epithelial (TCMK-1) cells were treated with50 ng/ml TRAIL in the presence or absence of 30 μM eupaforin for 24 h. The cell morphology was examined using interference light microscopy. The level of apoptosis was assessed by measuring the sub-G1 fraction using flow cytometry. **C.** MC and TCMK1 cells were treated with the indicated concentrations of eupafolin for 24 h. The protein levels of Mcl-1, Bim and actin were determined by western blotting. The level of actin was used as the loading control.

Next, we investigated whether eupafolin and TRAIL could enhance apoptosis in other cancer cells and normal cells. As shown in Figure 5A, we found that combined treatment with eupafolin and TRAIL enhanced the sub-G1 population and PARP cleavage in U251MG (glioma cells) and DU145 cells (prostate cancer cells). Furthermore, eupafolin induced down-regulation of Mcl-1 expression in both cells. However, up-regulation of Bim expression was detected in DU145 cells, but not U251MG cells (Figure 5A). In contrast, eupafolin plus TRAIL had no effect on apoptosis in human mesangial cells (MC) and mouse renal tubular epithelial (TCMK-1) cells (Figure 5B). Interestingly, eupafolin did not change expression levels of Mcl-1 and Bim protein in both normal cells (Figure 5C). These data indicate that eupafolin induces TRAIL-mediated apoptosis in cancer cells, but not in normal cells.

### Combined treatment with eupafolin and TRAIL inhibits tumor growth *in vivo*

**Figure 6:**
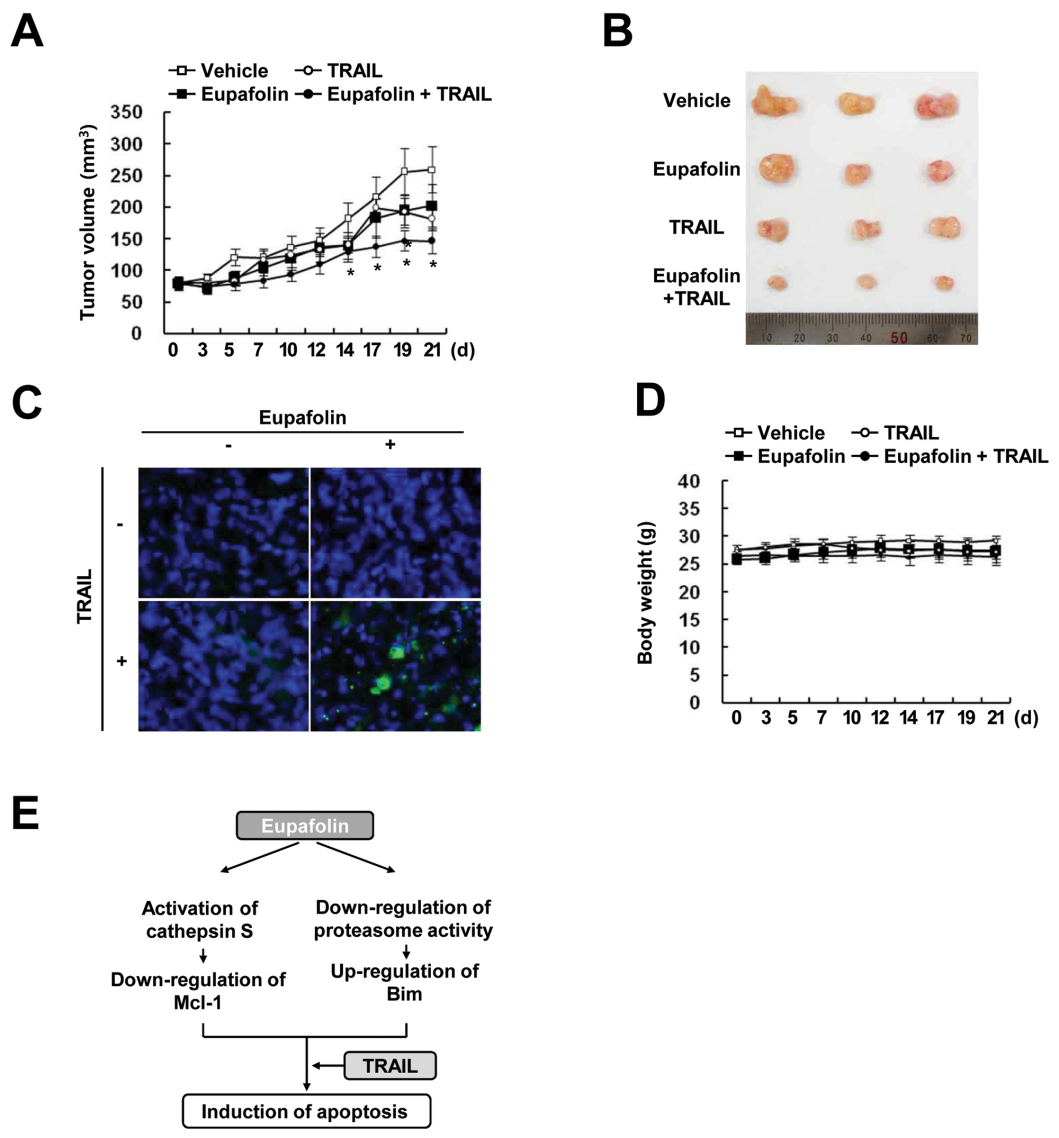
Tumor growth *in vivo* is reduced by the combined treatment with eupafolin and TRAIL Nude mice were subcutaneously inoculated with Caki cells. Tumor volume was monitored during the following treatments: vehicle, eupafolin (10 mg/kg; i.p.), GST-TRAIL (3 mg/kg, i.p.), or eupafolin plus TRAIL for 21 days. **A.** The graph shows changes in the tumor volume. Number of animals per group = 7. Data are means ± SE (n=7). * *p* < 0.05 compared to vehicle. **B.** The size of the dissected-out tumors is shown. **C.** Representative images of tumors sections that were analyzed by TUNEL assay. Nuclear staining was performed with DAPI. **D.** Bodyweight changes during the experiment. **E.** Schematic models of eupafolin-mediated TRAIL sensitization.

Next, we examined the anti-cancer effect of combined treatment with eupafolin and TRAIL *in vivo* xenograft model. Mice bearing tumors were treated with eupafolin alone, TRAIL alone, and combined with eupafolin and TRAIL. As shown in Figure 6A and B, combined treatment with eupafolin and TRAIL markedly suppressed tumor growth, compared with that of the vehicle, eupafolin alone, and TRAIL alone. TUNEL analysis also showed that eupafolin plus TRAIL induced cell death (Figure 6C). Eupafolin plus TRAIL treatment had no effect on the body weight (Figure 6D). These data suggest that combined treatment with eupafolin and TRAIL inhibits tumor growth and induces apoptosis *in vivo*.

## DISCUSSION

In this study, we demonstrated that eupafolin sensitizes TRAIL-mediated apoptosis in cancer cells. Eupafolin induced down-regulation of Mcl-1 expression in a cathepsin S-dependent manner. In addition, we found that eupafolin markedly induced Bim expression through AMPK-dependent inhibition of proteasome activity (Figure 6E). Furthermore, combined treatment with eupafolin and TRAIL reduced the tumor volume and increased apoptosis in a xenograft model. Therefore, these data suggest that eupafolin could be an effective TRAIL sensitizer.

One of the mechanisms by which eupafolin-mediated TRAIL sensitization was the down-regulation of Mcl-1 expression. As shown in Figure 3A and 3B, eupafolin inhibited Mcl-1 expression at the post-translational levels via modulation of protein stability. Since Mcl-1 is mainly degraded by the ubiquitin-proteasome pathway [16, 17], we tested proteasome inhibitors could reverse eupafolin-mediated down-regulation of Mcl-1. However, proteasome inhibitor (lactacystin) had no effect on Mcl-1 expression in eupafolin-treated cells (Figure 3C). Recently, it is reported that Mcl-1 is also regulated by lysosome-dependent protein degradation. For examples, cathepsin B degrades Mcl-1 proteins, resulting in apoptosis of FTY720-treated leukemia cells [23], and YM155 also induced down-regulation of Mcl-1 expression via lysosome-dependent manner in human oral cancer cells [24]. In our study, eupafolin-induced down-regulation of Mcl-1 expression was reversed by cathepsin S and cathepsin B inhibitor (Figure 3E). However, while knock-down of cathepsin B has no effect on Mcl-1 expression, but knock-down of cathepsin S rescued inhibition of Mcl-1 expression in eupafolin-treated cells. Therefore, our data suggested that eupafolin-mediated down-regulation of Mcl-1 is dependent of cathepsin S.

The other mechanism of eupafolin-mediated TRAIL sensitization is up-regulation of Bim expression. Eupafolin-mediated up-regulation of Bim is also regulated at the post-translational level. Eupafolin inhibited proteasome activity, and induced ubiquitination of proteins (Figure 4E and 4F). However, we found that expression levels of three critical proteasome subunits (PSMA5, PSMB5 and PSMD4/S5a) did not change in eupafolin-treated cells. (Figure 4G and 4H). Previous studies reported that degradation of Bim protein is modulated by E3 ligase, such as tripartite motif-containing protein 2 (TRIM2) [25] and Cbl [26]. Therefore, we also investigated that eupafolin induced TRIM2 and Cbl expression, but expression of both E3 ligases was not changed by eupafolin (negative data not shown). In our study, eupafolin increased AMPK phosphorylation (Figure 4I), and AMPK inhibitor and knock-down of AMPK by siRNA inhibited eupafolin-induced Bim expression (Figure 4J and 4K). Concannon et al., reported that AMPK-mediated Bim activation is related with excitotoxic stress-induced apoptosis. Excitotoxic stress-induced ATP depletion, resulted in AMPK activation, and then increased Bim expression, but not other BH3 proteins (PUMA and Bid) [21]. The zinc also induces neuronal death via AMPK-dependent up-regulation of Bim expression [22]. Angiotensin II induced apoptosis through E2F1-mediated up-regulation of Bim expression by activation of AMPK [27]. However, these papers suggested that AMPK induced Bim expression at the transcriptional levels. In our studies, AMPK regulated Bim expression at the post-translational levels (Figure 4B). Therefore, eupafolin-induced up-regulation of Bim expression by AMPK activation might be associated with other mechanisms. The other function of AMPK is a modulation of proteasome activity. For example, Xu et al. reported that AMPK inhibition induced 26S proteasome activity via induction of 26S proteasome assembly [28]. In our study, AMPK activator (AICAR) reduced proteasome activity (Figure 4L). Therefore, our data indicated that eupafolin induced Bim expression at the post-translational level via AMPK-dependent modulation of proteasome activity.

Collectively, these findings suggest that eupafolin sensitizes TRAIL-mediated apoptosis through the down-regulation of Mcl-1 expression and up-regulation of Bim expression in the human renal Caki cells. Therefore, eupafolin could be an attractive sensitizer for TRAIL resistance cancer cells.

## MATERIALS AND METHODS

### Cell cultures and materials

Human renal carcinoma cells (Caki), human glioma cells (U251MG) and human prostate cancer cells (DU145) were obtained from the American Type Culture Collection (Manassas, VA, USA). The mouse kidney (TCMK-1, ATCC-CCL-139) cells were obtained from the American Type Culture Collection (Manassas, VA, USA). Normal human mesangial cells (NHMCs) were purchased from Lonza (CC-2559, Basel, Switzerland). The culture medium used throughout these experiments was Dulbecco's modified Eagle's medium (DMEM) containing 10% fetal bovine serum (FBS), 20 mM HEPES buffer and 100 μg/ml gentamicin. PCR primers were purchased from Macrogen (Seoul, Korea). GST-TRAIL cDNA plasmid was a gift from Dr. Kim YS (Ajou university, Korea). Eupafolin was purchased Indofine (Hillsborough Township, NJ, USA). Recombinant human TRAIL and z-VAD-fmk was purchased from R&D system (Minneapolis, MN, USA). Cathepsin S inhibitor (Z-FL-COCHO) and compound C was purchased from Calbiochem (Darmstadt, Germany). E64D was purchased from Cayman chemical (Ann Arbor, MI, USA). Pepstatin A was purchased from ENZO (Ann Arbor, MI, USA). Anti-Mcl-1 (sc-819), anti-Bcl-xL (sc-634), anti-Bcl-2 (sc-783), anti-cIAP2 (sc-7944), anti-Cox IV (sc-292052), anti-ubiquitin (sc-8017), anti-cathepsin S (sc-30057), anti-cathepsin B (sc-13985) and anti-AMPK (sc-25792) antibodies were purchased from Santa Cruz Biotechnology (Santa Cruz, CA, USA). Anti-Bim (AB17003) was purchased from Millipore Corporation (Billerica, MA, USA). Anti-caspase-3 (ADI-AAP-113) antibody was purchased from ENZO (Ann Arbor, MI, USA). Anti-cytochrome c (cat. 556433), anti-Bax (cat. 556467), anti-FADD (cat. 556402) and anti-XIAP (cat.610762) antibodies were purchased from BD Biosciences (San Jose, CA, USA). Anti-PARP (#9542), anti-cleaved caspase-3 (9661S), anti-DR5 (8074S), anti-cIAP1 (4952S), anti-PSMA5 (#2457), anti-PSMB5 (#11903), anti-PSMD4/S5a (#12441) and anti-p-AMPK (#2531) antibodies were purchased from Cell Signaling Technology (Beverly, MA, USA). Anti-actin (A5441) antibody was obtained from Sigma– Aldrich (St. Louis, MO, USA). Other reagents were purchased from Sigma Chemical Co. (St. Louis, MO, USA).

### Flow cytometry analysis

For flow cytometry, the cells were resuspended in 100 μL of phosphate-buffered saline (PBS), and 200 μL of 95% ethanol was added while the cells were being vortexed. Then, the cells were incubated at 4°C for 1 h, washed with PBS, resuspended in 250 μL of 1.12% sodium citrate buffer (pH 8.4) with 12.5 μg of RNase and incubated for an additional 30 min at 37°C. The cellular DNA was then stained by adding 250 μL of a propidium iodide solution (50 μg/mL) to the cells for 30 min at room temperature. The stained cells were analyzed by fluorescent-activated cell sorting on a FACS can flow cytometer to determine the relative DNA content, which was based on the red fluorescence intensity.

### Western blot analysis

Cells were washed with cold PBS and lysed on ice in 50 μL of lysis buffer (50 mM Tris–HCl, 1 mM EGTA, 1% Triton X-100, 1 mM phenylmethylsulfonyl fluoride, pH 7.5). Lysates were centrifuged at 10,000 g for 15 min at 4°C, and the supernatant fractions were collected. Proteins were separated by SDS–PAGE and transferred to an Immobilon-P membrane. Specific proteins were detected using an enhanced chemiluminescence (ECL) Western blot kit according to the manufacturer's instructions.

### 4′,6′-Diamidino-2-phenylindole staining (DAPI) for nuclei condensation and fragmentation

To examine cellular nuclei, the cells were fixed with 1% paraformaldehyde on glass slides for 30 min at room temperature. After the fixation, the cells were washed with PBS and a 300 nM 4′, 6′-diamidino-2-phenylindole solution (Roche, Mannheim, Germany) was added to the fixed cells for 5 min. After the nuclei were stained, the cells were examined by fluorescence microscopy.

### Cell death assessment by DNA fragmentation assay

The cell death detection ELISA plus kit (Boehringer Mannheim, Indianapolis, IN, USA) was used for assessing apoptotic activity by detecting fragmented DNA within the nucleus in eupafolin alone, TRAIL alone, and combination with eupafolin and TRAIL-treated cells. Briefly, each culture plate was centrifuged for 10 min at 200 g, the supernatant was removed, and the pellet was lysed for 30 min. After centrifuging the plate again at 200 g for 10 min, and the supernatant that contained the cytoplasmic histone-associated DNA fragments was collected and incubated with an immobilized anti-histone antibody. The reaction products were incubated with a peroxidase substrate for 5 min and measured by spectrophotometry at 405 and 490 nm (reference wavelength) with a microplate reader. The signals in the wells containing the substrate alone were subtracted as the background.

### Determination of synergy

The possible synergistic effect of eupafolin and TRAIL was evaluated using the isobologram method. In brief, cells were treated with different concentrations of eupafolin and TRAIL alone or in combination. After 24 h, relative survival was assessed, and the concentration effect curves were used to determine the IC50 (the half-maximal inhibitory concentration) values for each drug alone and in combination with a fixed concentration of the second agent.

### Asp-Glu-Val-Asp-ase (DEVDase) activity assay

To evaluate DEVDase activity, cell lysates were prepared after their respective treatments with TRAIL in the presence or absence of eupafolin. Assays were performed in 96-well microtiter plates by incubating 20 μg of cell lysates in 100 μL of reaction buffer (1% NP-40, 20 mM Tris-HCl, pH 7.5, 137 mM NaCl, 10% glycerol) containing a caspase substrate [Asp-Glu-Val-Asp-chromophore-p-nitroanilide (DVAD-pNA)] at 5 μM. Lysates were incubated at 37°C for 2 h. Thereafter, the absorbance at 405 nm was measured with a spectrophotometer.

### Determination for the mitochondrial membrane potential by rhodamine 123

Rhodamine 123 (Molecular Probes Inc., Eugene, OR, USA) uptake by mitochondria is directly proportional to its membrane potential. After treatment, cells were incubated with rhodamine 123 (5 μM) for 5 min in the dark at 37°C. The cells were harvested and suspended in PBS. The mitochondrial membrane potential was subsequently analyzed using a flow cytometer (Becton–Dickinson, Franklin Lakes, NJ, USA).

### Analysis of cytochrome *c* release

The cells were harvested, washed once with ice-cold PBS and gently lysed for 2 min in 80 μL ice-cold lysis buffer [250 mM sucrose, 1 mM EDTA, 20 mM Tris–HCl (pH 7.2), 1 mM DTT, 10 mM KCl, 1.5 mM MgCl2, 5 μg/mL pepstatin A, 10 μg/mL leupeptin and 2 μg/mL aprotinin]. Lysates were centrifuged at 12,000 g at 4°C for 10 min to obtain the supernatants (cytosolic extracts free of mitochondria) and the pellets (fraction that contains mitochondria). The resulting cytosolic fractions were used for Western blot analysis with an anti-cytochrome c antibody.

### Reverse transcription polymerase chain reaction (RT-PCR) and quantitative PCR (qPCR)

Total RNA was isolated using the TriZol reagent (Life Technologies, Gaithersburg, MD, USA), and cDNA was prepared using M-MLV reverse transcriptase (Gibco-BRL, Gaithersburg, MD, USA) according to the manufacturer's instructions. The following primers were used for the amplification of human Mcl-1, Bim, PSMA5, PSMB5, PSMD4/S5a and actin: Mcl-1 (forward) 5′-GCG ACT GGC AAA GCT TGG CCT CAA-3′ and (reverse) 5′-GTT ACA GCT TGG ATC CCA ACT GCA-3′; Bim (forward) 5′-ATG GCA AAG CAA CCT TCT GA-3′ and (reverse) 5′-CTG TCT GTG TCA AAA GAG-3′; PSMA5 (forward) 5′-TAT CAA GCT TGG TTC TAC AG-3′ and (reverse) 5′-ATG TGA GCA TCA ATC TCT AC-3′; PSMB5 (forward) 5′-TGT AGC AGC TGC CTC CAA AC-3′ and (reverse) 5′-CCC TGA AAT CCG GTT CCC TT-3′; PSMD4/S5a (forward) 5′-CTA CAT ACT GTC CAA CCC AA-3′ and (reverse) 5′-AGT TTC ACC AGA TCC TTC TC-3′; and actin (forward) 5′-GGC ATC GTC ACC AAC TGG GAC-3′ and (reverse) 5′-CGA TTT CCC GCT CGG CCG TGG-3′. The PCR amplification was carried out using the following cycling conditions: 94°C for 3 min followed by 17 (actin) or 28 cycles (Mcl-1 and Bim) of 94°C for 40 s, 56°C for 40 s, 72°C for 1 min, and a final extension at 72°C for 5 min. The amplified products were separated by electrophoresis on a 1.5% agarose gel and detected under UV light. For qPCR, cDNA and forward/reverse primers (200 nM) were added to 2 × KAPA SYBR Fast master mix, and reactions were performed on LightCycler 480 real-time amplification instrument (Roche, Basel, Switzerland). The following primers were used for the amplification of human Mcl-1, Bim and actin: Mcl-1 (forward) 5′-ATG CTT CGG AAA CTG GAC AT-3′ and (reverse) 5′-TCC TGA TGC CAC CTT CTA GG-3′; Bim (forward) 5′-CAC AAA ACC CCA AGT CCT CCT T-3′ and (reverse) 5′-TTC AGC CTG CCT CAT GGA A-3′; and actin (forward) 5′-CTA CAA TGA GCT GCG TGT G-3′ and (reverse) 5′-TGG GGT GTT GAA GGT CTC-3′. Threshold cycle number (Ct) of each gene was calculated, and actin was used as reference genes. Delta-delta Ct values of genes were presented as relative fold induction [29].

### Small-interfering RNAs (siRNAs)

The GFP (control), cathepsin B and AMPK siRNA duplexes used in this study were purchased from Santa Cruz Biotechnology (Santa Cruz, CA, USA), cathepsin S siRNA duplexes is purchased from Dharmacon (Lafayette, CO, USA), and Bim siRNA duplexes is obtained from Bioneer (Dejeon, Korea). Cells were transfected with siRNA oligonucleotides using Oligofectamine reagent (Invitrogen, Carlsbad, CA, USA) according to the manufacturer's recommendations.

### Mcl-1 constructs and stable cell

The Caki cells were transfected in a stable manner with the pFLAG-CMV4/Mcl-1 plasmid or control plasmid pcDNA 3.1 vector using Lipofectamine2000 as prescribed by the manufacturer (Invitrogen, Carlsbad, CA). After 48 h of incubation, transfected cells were selected in primary cell culture medium containing 700 μg/mL G418 (Invitrogen, Carlsbad, CA). After 2 or 3 weeks, single independent clones were randomly isolated, and each individual clone was plated separately. After clonal expansion, cells from each independent clone were tested for Mcl-1 expression by immunoblotting.

### Proteasome activity assay

Fluorescence-based proteasome activity was assessed with ZsGreen (proteasome sensor vector) stably transfected Caki cell lines. Relative fluorescence activity was analyzed by using a FACS Canto^TM^ (BD Biosciences, San Jose, CA, USA).

### Animal

Male BALB/c-nude mice, aged 5 weeks, were purchased from the Central Lab Animal Inc. (Seoul, Korea). All the mice were allowed 1 week to acclimatize to the surroundings before the experiments, and were kept at 25 ± 2°C, with a relative humidity of 55 ± 5% and a 12 h light–dark cycle. The study protocol was approved by the IRB Keimyung University Ethics Committee (KM-2013-82).

### *In vivo* xenograft model

Each mouse was subcutaneously (s.c.) injected on each flank with Caki cells (2 × 10^6^). After tumors had grown after approximately 2 weeks, 28 mice were randomly divided in to four treatment groups: (1) vehicle alone, (2) eupafolin alone, (3) GST-TRAIL alone, and (4) eupafolin plus GST-TRAIL. Eupafolin and GST-TRAIL were administered at 10 mg/kg and 3 mg/kg, respectively. Eupafolin and GST-TRIAL were prepared in PBS (pH 7.4). The mice received an intraperitoneal (i.p.) injection of eupafolin and GST-TRAIL. Treatment was administered three times a week for 3 weeks. The tumor size was measured three times a week using a Vernier's caliper (Mytutoyo Co., Tokyo, Japan) to measure two perpendicular diameters, and the tumor size was calculated using the equation (length × width^2^)/2. The animals were sacrificed by cervical dislocation, and the tumors were collected for histological analysis. The tumors were fixed in 30% formalin, embedded in OCT compound (Miles Inc., Elkhart, IN, USA) and cut into 20-μm sections using a cryostat (SLEE International Inc., duarte, CA, USA).

### TUNEL assay

Apoptosis in tumor cells was detected by terminal deoxynucleotide transferase (TdT)-mediated dUTP nick-end labeling (TUNEL) assay [30]. It was performed using the ApopTag Fluorescein *In Situ* Apoptosis Detection Kit (Millipore) as the manufacturer's protocol.

### Statistical analysis

The data were analyzed using a one-way ANOVA and post-hoc comparisons (Student-Newman-Keuls) using the Statistical Package for Social Sciences 22.0 software (SPSS Inc., Chicago, IL).

## SUPPLEMENTARY MATERIAL FIGURE


