# Supplementary material for: Eupafolin enhances TRAIL-mediated apoptosis through cathepsin S-induced down-regulation of Mcl-1 expression and AMPK-mediated Bim up-regulation in renal carcinoma Caki cells
Source: Oncotarget. 2016 Aug 25;7(40):65707–20. doi: 10.18632/oncotarget.11604 (PMC5323186; doi:10.18632/oncotarget.11604)
Supplement: Supplementary file 1 [file oncotarget-07-65707-s001.pdf]

Eupafolin enhances TRAIL-mediated apoptosis through cathepsin S-induced down-regulation of Mcl-1 expression and AMPK-mediated Bim up-regulation in renal carcinoma Caki cells

SUPPLEMENTARY FIGURE

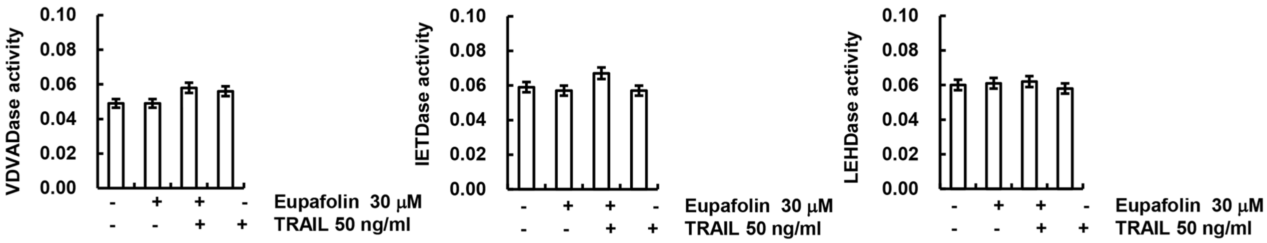

Supplementary Figure S1: Effect of combined treatment with eupafolin and TRAIL on various caspases activity. Caki cells were treated with 50 ng/ml TRAIL in the presence or absence of 30 μM eupafolin for 24 h. Caspase activities were determined with colorimetric assays using caspase-2 (VDVADase), caspase-8 (IETDase) and caspase-9 (LEHDase) assay kits. The values in figure represent the mean ± SD from three independent samples.
